# Invasive aspergillosis infection in an immunocompromised patient

**DOI:** 10.1590/0037-8682-0540-2023

**Published:** 2024-02-05

**Authors:** Elif Gündoğdu, Nevin Aydın

**Affiliations:** 1 Eskişehir Osmangazi University, Faculty of Medicine, Department of Radiology, Eskişehir, Turkey.

**FIGURE 1:**
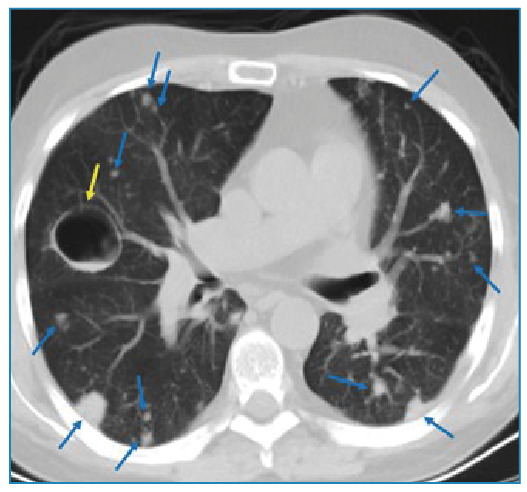
Thoracic CT showing cavitary (yellow arrow) and multiple solid nodules in both lungs (blue arrows).

**FIGURE 2:**
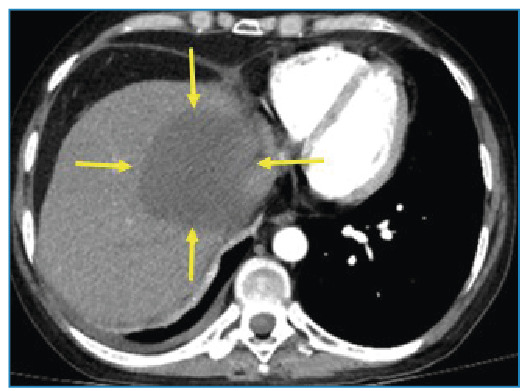
Axial plane CT showing large solitary hypodense lesion in liver parenchyma (yellow arrows).

**FIGURE 3:**
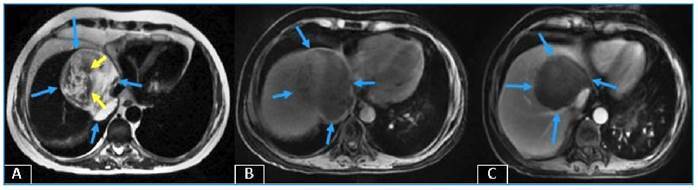
MRI showing **(A)** heterogeneous (containing hypointense areas: yellow arrows) hyperintense lesion on T2-weighted image (blue arrows), **(B)** hypointense on T1-weighted image (blue arrows), and **(C)** no contrast enhancement on post-contrast T1-weighted image (blue arrows).

A 40-year-old male patient was referred to our radiology department complaining of chest pain, fever, and sputum. His medical history included acute lymphoblastic leukemia, bone marrow transplantation, and graft-versus-host disease. Laboratory studies showed a total leukocyte count of 14.2×10^3^ uL (92.9% of neutrophils), with raised procalcitonin (0.44 ng/ml) and C-reactive protein (75.9 mg/L) levels. Thoracic CT revealed multiple nodules and masses, some in cavitary form, dispersed in both lungs (Figure 1). Sputum culture yielded *Aspergillus fumigatus* and *flavus*. At follow-up for invasive pulmonary aspergillosis, a newly developed hypodense lesion was detected in the liver parenchyma on control thoracic CT (Figure 2). MRI revealed a heterogeneous (due to hypointense areas) hyperintense lesion on T2-weighted image and a hypointense non-enhancing lesion on T1-weighted images (Figure 3). Aspergillosis was confirmed histopathologically. Fungal infections such as invasive aspergillosis are common in patients with severely compromised immune systems, including those with neutropenia, hematologic malignancies, organ transplants, HIV/AIDS, or long-term corticosteroid use^1^ ^,^ ^2^. Because of inhalation transmission, the lungs are the most commonly affected organs^3^. Liver *Aspergillus* has rarely been reported in case reports^2^. Importantly, although rare, the liver may also be affected in patients with risk factors.
